# Social support following diagnosis and treatment for colorectal cancer and associations with health‐related quality of life: Results from the UK ColoREctal Wellbeing (CREW) cohort study

**DOI:** 10.1002/pon.4556

**Published:** 2017-11-01

**Authors:** Joanne Haviland, Samantha Sodergren, Lynn Calman, Jessica Corner, Amy Din, Deborah Fenlon, Chloe Grimmett, Alison Richardson, Peter W. Smith, Jane Winter, members of Study Advisory Committee, Claire Foster

**Affiliations:** ^1^ Institute of Cancer Research Clinical Trials and Statistics Unit, Division of Clinical Studies The Institute of Cancer Research London UK; ^2^ Macmillan Survivorship Research Group, Faculty of Health Sciences University of Southampton Southampton UK; ^3^ Executive Office University of Nottingham Nottingham UK; ^4^ College of Health and Human Sciences Swansea University, Swansea Wales UK; ^5^ Faculty of Health Sciences University of Southampton Southampton UK; ^6^ University Hospital Southampton NHS Foundation Trust Southampton UK; ^7^ Social Statistics and Demography, Social Sciences University of Southampton Southampton UK

**Keywords:** cancer survivorship, colorectal cancer, health‐related quality of life, oncology, social support

## Abstract

**Objective:**

Social support is acknowledged as important in cancer survivorship, but little is known about change in support after cancer diagnosis and factors associated with this, particularly in colorectal cancer. The CREW cohort study investigated social support up to 2 years following curative intent surgery for colorectal cancer.

**Methods:**

A total of 871 adults recruited pre‐treatment from 29 UK centres 2010 to 2012 consented to follow‐up. Questionnaires at baseline, 3, 9, 15, and 24 months post‐surgery included assessments of social support (Medical Outcomes Study‐Social Support Survey, MOS‐SSS) and health‐related quality of life (HRQoL). Socio‐demographic, clinical and treatment details were collected. Longitudinal analyses assessed social support over follow‐up, associations with participant characteristics, and HRQoL.

**Results:**

Around 20% were living alone and 30% without a partner. Perceived social support declined in around 29% of participants, with 8% of these reporting very low levels overall from baseline to 2 years (mean MOS‐SSS overall score < 40 on a scale from 0 to 100). Older age, female gender, greater neighbourhood deprivation, presence of co‐morbidities, and rectal cancer site were significantly associated with reductions in perceived support. Poorer HRQoL outcomes (generic health/QoL, reduced wellbeing, anxiety, and depression) were significantly associated with lower levels of social support.

**Conclusions:**

Levels of social support decline following colorectal cancer diagnosis and treatment in nearly a third of patients and are an important risk factor for recovery of HRQoL. Assessment of support early on and throughout follow‐up would enable targeted interventions to improve recovery, particularly in the more vulnerable patient groups at risk of poorer social support.

## 1 BACKGROUND

Social support is widely regarded as beneficial to people living with and beyond cancer, particularly for psychological wellbeing,^1^ and has been proposed as an important aspect of recovery,^2^ as well as in planning survivorship care.^3^ Higher levels of support have been shown to be associated with better health outcomes and health‐related quality of life (HRQoL) following cancer treatment, with most studies in breast cancer.^4^, ^5^ However, few studies have investigated the role of social support in colorectal cancer, despite being the most common cancer type that affects both genders. Results from small cross‐sectional studies in colorectal cancer have shown associations between lower social support and poorer psychological wellbeing and HRQoL.^6^, ^7^, ^8^ Longitudinal studies showing similar associations are limited by short or incomplete follow‐up (≤ 1 year) or no pre‐treatment baseline data.^9^, ^10^, ^11^, ^12^

Furthermore, there is very little published evidence on changing levels of social support following a cancer diagnosis. Studies in breast cancer suggest that support levels decline following diagnosis and treatment,^13^, ^14^, ^15^, ^16^ although 1 study reported levels remaining moderately high over 3 years' follow‐up.^5^ Limited evidence from other cancer sites also varies; support levels were reported to be stable up to 1 year follow‐up in a prostate cancer study (*N* = 134,^17^), but declined pre‐treatment to post‐treatment in head and neck cancer (*N* = 32^18^). Longitudinal data on social support in colorectal cancer are extremely sparse; a study in China (*N* = 227) reported declining support up to 1 year following surgery, particularly in women and those with a lower family income.^19^

Alternative supportive self‐management models for cancer after‐care are being implemented in the UK, such as patient‐triggered follow‐up whereby patients initiate contact if they experience symptoms or have concerns. Compared with the routine follow‐up approach, patients are likely to have less contact with health care professionals, and therefore it is more important than ever to determine patterns of social support following treatment. Identification at diagnosis of individuals who might be at greater risk of poor and declining levels of support as well as the potential effects of this on recovery of HRQoL will help tailor management packages for these patients.

The UK ColoREctal Wellbeing study (CREW) is a large‐scale cohort study investigating factors associated with recovery of health and wellbeing following colorectal cancer. The domains of assessment were informed by a conceptual framework of recovery following cancer diagnosis and treatment,^2^ which hypothesised that a number of factors including social support would affect recovery. The main aim of this paper was to describe patient‐reported social support pre‐surgery up to 2 years' follow‐up, in particular to investigate any change in support over time and which individuals were more likely to report poorer levels of support. A secondary aim was to investigate associations between social support over follow‐up and HRQoL outcomes.

## 2 METHODS

### 2.1 Study design and participants

CREW is a multicentre, prospective cohort study of adults with non‐metastatic colorectal cancer. Details of eligibility criteria, recruitment strategy, and sample size are provided elsewhere.^20^ In brief, eligible individuals were approached before primary surgery from 29 UK cancer centres between November 2010 and March 2012. Written consent was obtained, and baseline questionnaires completed prior to surgery whenever possible. Follow‐up questionnaires were completed at 3, 9, 15, and 24 months post‐surgery (longer‐term follow‐up is ongoing). Socio‐demographic information was also collected at consent. Participants reported whether they lived alone, and self‐reported co‐morbidities were recorded from 3 months onwards; clinical and treatment details were taken from medical notes. Ethical approval was granted by the UK NHS NRES Committee South Central—Oxford B (REC ref: 10/H0605/31).

### 2.2 Measures

Full details of the questionnaire measures used in the CREW study are provided elsewhere.^20^ The measures presented in this paper are described in brief:

The Index of Multiple Deprivation^21^ is the official measure of relative deprivation for small areas in England and uses postcodes to calculate an overall deprivation score based on 7 weighted domains of deprivation including income, employment, education, health, crime, barriers to housing and services and living environment.

Availability of social support was assessed in various ways: participants reported whether or not they lived alone, and number of close friends and family. The MOS Social Support Survey (MOS‐SSS^22^) yields an overall measure of social support (mean of all 19 items), subscales representing emotional/informational support, tangible support, affectionate support and positive social interaction (higher scores represent greater support, range 0–100), and an individual item relating to the extent to which participants feel they have “someone to do things with to help you get your mind off things” (Appendix S1).

The EQ‐5D^23^ measures generic health status/QoL, comprising 5 domains (mobility, self‐care, usual activities, pain/discomfort, anxiety/depression), each scored as none/some/severe problems, which can be summarised overall as presence/absence of problems on ≥1 of the domains.

The Personal Wellbeing Index—Adult (PWI‐A^24^) contains 8 items of satisfaction corresponding to standard of living, health, achieving in life, relationships, safety, community‐connectedness, future security, and spirituality/religion. A higher overall score of wellbeing denotes better wellbeing (range 0–100; < 70 represents reduced wellbeing).

Anxiety and depression were assessed using the State‐Trait Anxiety Inventory (STAI^25^) and the Centre for Epidemiologic Studies Depression Scale (CES‐D^26^). Higher scores indicate greater anxiety and depression; ≥40 indicates clinical levels of anxiety^27^ (range 20–80); and ≥20 has been suggested to indicate clinical depression for cancer patients (Katz et al, 2004,^28^ range 0–60).

### 2.3 Statistical methods

Published guidance for missing items in subscales were applied where available; otherwise, if ≥75% of items had been completed, mean scores were imputed from completed items. MOS‐SSS scores were calculated according to published guidelines; binary variables were also created indicating whether a participant had responded “none” or “a little of the time” to all items within a subscale (versus “some/most/all of the time”). The Index of Multiple Deprivation was categorised into quintiles. Descriptive analyses summarised measures of social support at each time‐point. A sensitivity analysis was performed including only those questionnaires completed within specific timeframes around the expected date; as this made very little difference to the results, all data were included for the analyses presented.

For longitudinal analyses, length of follow‐up was calculated from surgery to date of questionnaire completion (or date questionnaire received in research office if unknown); timing of baseline questionnaire (pre/post‐surgery) was adjusted for in all regression models. Patterns of change in levels of social support from baseline to 2 years were analysed using: (1) Generalised Estimating Equations (GEE^29^) to assess mean levels of the individual domains of support for the overall cohort over follow‐up, and (2) group‐based trajectory analyses^30^ to investigate whether there were subgroups with distinct levels of support from the MOS‐SSS overall social support score. In brief, each individual participant has an observed trajectory; the modelling technique sorts the individual trajectories into clusters according to mean‐level changes in the outcome variable and rank orders these groups over follow‐up. The optimal number of distinct trajectories was determined using the Bayesian information criterion (BIC)^31^ to compare model fit (change in BIC >10 supports the more complex model), while aiming to avoid trajectories containing few individuals. The shape of each trajectory was assessed to determine whether it was best described by a linear, quadratic, or cubic function according to the significance of each term. Estimated proportions of participants within each trajectory were obtained, with 95% confidence intervals (CI). The estimated trajectory groups were plotted according to mean scores of MOS‐SSS overall social support score at each time‐point.

Predictors of group membership for the MOS‐SSS overall social support score were investigated by fitting baseline participant characteristics (socio‐demographic and clinical) in the trajectory models. Frequencies of participant characteristics in each group were compared with the reference group (best levels of support). Factors found to be significant or borderline significant (*P* < 0.1) from univariate analyses were modelled together, and only those which remained statistically significant for at least 1 of the group comparisons were retained in the final prediction models.

Associations between social support and HRQoL outcomes from baseline to 24 months (generic health/QoL measured by the EQ‐5D, personal wellbeing, clinical levels of anxiety and clinical levels of depression, all fitted as binary variables) were assessed in separate GEE models for each measure of social support (fitted as a time‐dependent variable to allow for repeated measures over follow‐up), adjusting for baseline participant characteristics. Odds ratios (OR) were used to describe the relative increase in odds of each HRQoL binary outcome with a unit increase in the measure of social support for MOS‐SSS overall social support score, and for each category compared with the reference category for the binary social support variables. The potential for a random effect of recruiting site was explored in the GEE models for HRQOL outcomes and was found to be negligible.

The Wald test was used to assess significance in all regression analyses. Statistical analyses were done using Stata version 14 and IBM Statistics SPSS version 24.

## 3 RESULTS

### 3.1 Characteristics of the sample

A total of 857 participants consented to follow‐up excluding 15 who withdrew at baseline. Response rates were 89% at baseline, 84% at 3 months, 82% at 9 months, 80% at 15 months, and 74% at 24 months. Baseline questionnaires were completed prior to primary surgery in 70% of participants, and within 3 months after surgery by a further 26%; reasons for post‐surgical baseline questionnaires included admission for emergency surgery. Participants with and without a 24‐month questionnaire were broadly similar in terms of demographic and clinical characteristics, with similar levels of social support at baseline. The mean age of participants at study entry was 68 years, with 60% male. The sample comprised 65% colon and 35% rectal cancer, disease stage was 14% Duke's A, 53% Duke's B, and 32% Duke's C. By 2 years, 79 participants had experienced a recurrence, 65 had died, and 105 had withdrawn for reasons such as a deterioration in health, co‐morbidities, significant life events, or that the participant felt the questions were no longer relevant as they had recovered from their cancer. Full details of participants are described elsewhere.^32^

### 3.2 Levels of social support from baseline to 2 years after surgery

At baseline, 70.6% of participants were married or living with a partner. Around 20% of participants reported that they lived alone (21.3% at 3 months and 23.5% at 24 months). The median number of close friends and family reported at baseline was 6 and 7, respectively, and remained stable over follow‐up. At baseline, 7.8% of participants reported that they had “someone to help them get their mind off things” none/a little of the time, increasing to 17.3% at 2 years (Table 1). Proportions reporting none/a little of the time to all items within the MOS‐SSS subscales at baseline and 2 years were 2.5% and 10.1% for emotional/informational support, 5.1% and 12.4% for tangible support, 4.5% and 12.9% for affectionate support, and 6.5% and 12.4% for positive social interaction. All of the MOS‐SSS subscales and the overall measure of social support indicated a statistically significant decrease in support over follow‐up (*P* < 0.001 for all MOS‐SSS scales) (Table 1), although the absolute change in mean scores was small for some domains, bordering on clinically important differences (effect size = 0.4 for change in overall social support score from baseline to 2 years).

**Table 1:** Levels of social support from baseline to 2 years

Measure of social support	Baseline	3 months	9 months	15 months	24 months
	*N* = 756	*N* = 668	*N* = 623	*N* = 579	*N* = 514
Living alone	N/A				
No		511 (76.5%)	469 (75.3%)	397 (65.6%)	365 (71.0%)
Yes		142 (21.3%)	142 (22.8%)	124 (21.4%)	121 (23.5%)
Unknown		15 (2.2%)	12 (1.9%)	58 (10.0%)	28 (5.4%)
Domestic status					
Married/living with partner	534 (70.6%)	478 (71.6%)	437 (70.1%)	372 (64.2%)	340 (66.1%)
Single/widowed/divorced/separated	218 (28.8%)	185 (27.7%)	185 (29.7%)	160 (27.6%)	149 (29.0%)
Unknown	4 (0.5%)	5 (0.7%)	1 (0.2%)	47 (8.1%)	25 (4.9%)
Number of close friends					
Median (IQR)	6 (3–10)	5 (3–10)	5 (3–8)	5 (3–10)	5 (3–9.75)
Number of close family members					
Median (IQR)	7 (4–10)	5 (4–10)	6 (3–10)	6 (3–10)	6 (4–10)
Medical Outcomes Study (MOS) Social Support Survey^a^					
Overall social support					
Mean (SD);	80.9 (20.9)	77.9 (23.2)	74.1 (25.4)	74.5 (26.2)	72.0 (27.3)
Emotional/informational support					
Mean (SD); Number (%) responding none/a little of the time to all items	78.3 (23.5) 20/753 (2.5%)	75.6 (25.2) 32/663 (4.8%)	71.3 (27.7) 39/618 (6.3%)	71.1 (29.0) 41/534 (7.7%)	68.8 (30.1) 51/504 (10.1%)
Tangible support					
Mean (SD); Number (%) responding none/a little of the time to all items	82.8 (25.9) 38/752 (5.1%)	80.2 (28.4) 46/660 (7.0%)	75.3 (32.3) 72/614 (11.7%)	76.4 (32.1) 60/536 (11.2%)	74.1 (32.8) 62/500 (12.4%)
Affectionate support					
Mean (SD); Number (%) responding none/a little of the time to all items	87.1 (24.1) 34/748 (4.5%)	83.7 (26.9) 44/661 (6.7%)	80.1 (29.7) 62/618 (10.0%)	80.6 (30.1) 56/534 (10.5%)	77.8 (31.6) 65/503 (12.9%)
Positive social interaction					
Mean (SD); Number (%) responding none/a little of the time to all items	79.6 (26.4) 48/743 (6.5%)	76.3 (28.2) 61/655 (9.3%)	74.5 (29.7) 76/617 (12.3%)	75.7 (29.4) 63/535 (11.8%)	72.7 (30.3) 62/500 (12.4%)
Someone to do things with to help you get your mind off things					
Number (%) responding none/a little of the time	56/719 (7.8%)	74/617 (12.0%)	92/580 (15.9%)	78/504 (15.5%)	85/492 (17.3%)

N/A, not available (not asked on questionnaire); SD, standard deviation.

a All MOS subscales can range from 0 to 100, with *higher* scores indicating *greater* support.

**Figure 1:**
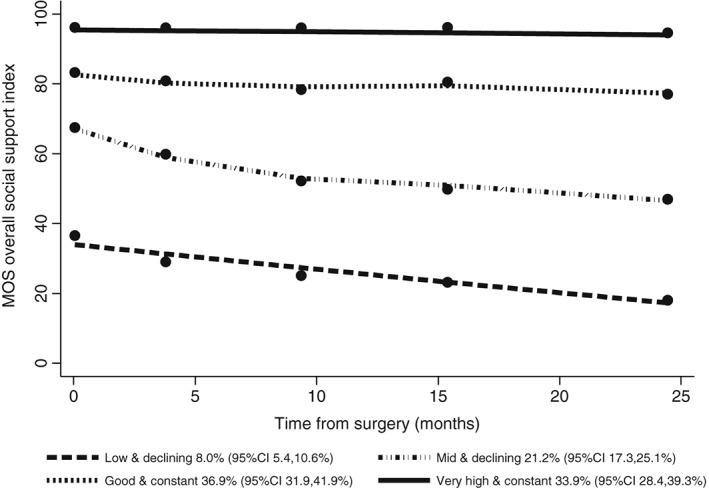
Estimated trajectories from baseline to 2 years' follow‐up for MOS‐SSS overall social support index (*N* = 808 with data available)

The optimal number of distinct trajectories identified for the MOS‐SSS overall social support score over follow‐up was 4, based on assessing the change in BIC (83.19 from 3 groups to 4) and estimated number of participants in the smallest group (8%). The 4 groups were as follows: (1) very high and constant levels of support, with an estimated 33.9% (95%CI 28.4–39.3%) of participants, (2) good and constant levels of support, 36.9% (95%CI 31.9–41.9%), (3) mid and declining levels of support, 21.2% (95%CI 17.3–25.1%), and (4) low and declining levels of support, 8.0% (95%CI 5.4–10.6%). The patterns of the groups are shown in Figure 1, which illustrates the decrease in mean levels of overall social support for Groups 3 and 4.

### 3.3 Associations between baseline participant characteristics and levels of social support over follow‐up

Participants estimated to be in Group 3 (mid/declining support) were significantly older (52% aged >70) and with a greater proportion of women (47%) compared with Group 1 (very high/constant support; 39% aged >70 and 36% women, *P* = 0.046 for both) (Table 2). For Group 4 (low/declining support), participants were significantly more likely to have higher neighbourhood deprivation (52% in fourth or fifth quintiles versus 37% for Group 1; *P* = 0.049), co‐morbidities (84% versus 69%, *P* = 0.021), and to have had rectal rather than colon cancer (42% versus 35%, *P* = 0.013). These factors remained statistically significant when included together in the final trajectory model for the MOS‐SSS overall score (Table 2). Odds ratios for trajectory group membership according to each participant characteristic in the final model are shown in Table A1 (Appendix). There were no independent significant associations between social support trajectory and Dukes stage, neoadjuvant or adjuvant therapy, or stoma, after allowing for the factors already identified, and no significant differences in participant characteristics between Groups 1 and 2, who had fairly constant levels of support over the 2 years.

**Table 2:** Associations between baseline characteristics and estimated trajectories of social support up to 2 years (odd ratio data are shown in Table A1)

Baseline characteristics	Trajectories of MOS‐SSS overall social support			
	Group 1 (very high and constant)	Group 2 (good and constant)	Group 3 (mid and declining)	Group 4 = (low and declining)
	*N* = 268 (%)	*N* = 308 (%)	*N* = 172 (%)	*N* = 60 (%)
Age	Ref. group	*P* = 0.392	*P* = 0.046	*P* = 0.460
≤60	39 (14.5)	73 (23.8)	33 (19.2)	14 (23.3)
61–70	126 (47.0)	113 (36.8)	50 (29.1)	16 (26.7)
71–80	83 (31.0)	86 (28.1)	61 (35.5)	21 (35.0)
>80	20 (7.5)	35 (11.4)	28 (16.3)	9 (15.0)
Unknown	0	1	0	0
Gender	Ref. group	*P* = 0.423	*P* = 0.046	*P* = 0.278
Male	171 (63.8)	184 (59.7)	91 (52.9)	35 (58.3)
Female	97 (36.2)	124 (40.3)	81 (47.1)	25 (41.7)
Neighbourhood deprivation quintile	Ref. group	*P* = 0.138	*P* = 0.114	*P* = 0.049
1^st^ (least deprived)	64 (24.2)	59 (19.7)	29 (17.3)	8 (13.3)
2^nd^	48 (18.2)	69 (23.0)	39 (23.2)	10 (16.7)
3^rd^	55 (20.8)	58 (19.3)	30 (17.9)	11 (18.3)
4^th^	50 (18.9)	46 (15.3)	34 (20.2)	20 (33.3)
5^th^ (most deprived)	47 (17.8)	68 (22.7)	36 (21.4)	11 (18.3)
Unknown	4	8	4	0
Any co‐morbidities	Ref. group	*P* = 0.176	*P* = 0.822	*P* = 0.021
No	70 (31.5)	65 (26.2)	41 (28.7)	7 (15.6)
Yes	152 (68.5)	183 (73.8)	102 (71.3)	38 (84.4)
Unknown^a^	46	60	29	15
Tumour site	Ref. group	*P* = 0.190	*P* = 0.995	*P* = 0.013
Colon	175 (65.3)	194 (63.6)	114 (66.3)	35 (58.3)
Rectum	93 (34.7)	111 (36.4)	58 (33.7)	25 (41.7)
Unknown	0	3	0	0

*P*‐values from Wald test comparing each Group with Group 1 (reference group) in multiple regression model including all variables in table and adjusting for pre/post‐surgery baseline.

a Co‐morbidities reported on 3‐month questionnaire.

### 3.4 Associations between levels of social support and HRQoL outcomes

Poorer HRQoL outcomes within 2 years were significantly associated with lower levels of social support throughout follow‐up, adjusting for baseline participant characteristics (Table 3). Poorer generic health/HRQoL (problems on ≥1 EQ‐5D domains) was significantly associated with lower overall support (*P* < 0.001), lack of affectionate support (*P* = 0.024), lack of positive social interaction (*P* < 0.001), and not having “someone to take their mind off things” (*P* = 0.008). Reduced personal wellbeing and high levels of depression were consistently significantly associated with lower levels of support (*P* < 0.001 for all MOS‐SSS domains). High levels of anxiety were significantly associated with lower levels of support (*P* < 0.001 for all domains except emotional/informational support where *P* = 0.018). Lack of positive social interaction appeared to have the greatest effect on the outcomes: odds ratio 2.55 (95%CI 1.61–4.04) for poorer generic health/QoL, 5.43 (3.79–7.79) for reduced personal wellbeing, 4.27 (2.93–6.22) for high levels of anxiety. and 5.65 (3.93–8.14) for high levels of depression.

**Table 3:** Associations between social support and HRQoL outcomes up to 2 years following surgery

Measure of social support from MOS‐SSS	Poorer generic health/QoL (EQ‐5D^a^)	Reduced personal wellbeing (PWI <70)	Clinical level anxiety (STAI ≥ 40)	Clinical level depression (CES‐D ≥ 20)
	OR^c^ (95%CI), *P*‐value	OR^c^ (95%CI), *P*‐value	OR^c^ (95%CI), *P*‐value	OR^c^ (95%CI), *P*‐value
Lower overall social support (total score)^b^	*P* < 0.001	*P* < 0.001	*P* < 0.001	*P* < 0.001
	1.01 (1.01,1.02)	1.04 (1.03,1.04)	1.02 (1.02,1.03)	1.03 (1.03,1.04)
Emotional/informational support	*P* = 0.064	*P* < 0.001	*P* = 0.018	*P* < 0.001
None/a little vs some/most/all of the time	1.64 (0.97,2.76)	3.38 (2.25,5.09)	1.64 (1.09,2.47)	2.70 (1.73,4.22)
Tangible support	*P* = 0.170	*P* < 0.001	*P* < 0.001	*P* < 0.001
None/a little vs some/most/all of the time	1.37 (0.87,2.16)	2.90 (2.00,4.20)	1.89 (1.33,2.70)	3.24 (2.24,4.69)
Affectionate support	*P* = 0.024	*P* < 0.001	*P* < 0.001	*P* < 0.001
None/a little vs some/most/all of the time	1.70 (1.07,2.71)	4.81 (3.26,7.11)	2.83 (1.88,4.26)	5.77 (4.00,8.32)
Positive social interaction	*P* < 0.001	*P* < 0.001	*P* < 0.001	*P* < 0.001
None/a little vs some/most/all of the time	2.55 (1.61,4.04)	5.43 (3.79,7.79)	4.27 (2.93,6.22)	5.65 (3.93,8.14)
Someone to do things with to help you get your mind off things	*P* = 0.008	*P* < 0.001	*P* < 0.001	*P* < 0.001
None/a little vs some/most/all of the time	1.68 (1.14,2.48)	3.71 (2.71,5.07)	2.92 (2.11,4.03)	4.04 (2.87,5.69)

CI, confidence interval; OR, odds ratio.

a Problems on ≥1 of the 5 EQ‐5D domains.

b Lower scores for MOS‐SSS overall social support indicate lower levels of support.

c Adjusting for baseline age, gender, deprivation, co‐morbidities, and tumour site.

## 4 DISCUSSION

### 4.1 Levels of social support

Our study found that, whilst the proportion of participants living alone and numbers of family and friends remained stable up to 2 years' follow‐up after surgery for colorectal cancer, levels of perceived social support significantly reduced across all the MOS‐SSS domains in the sample as a whole. For the overall sample, the proportion of participants reporting a lack of support in the individual domains more than doubled from baseline to 2 years. MOS‐SSS domain scores were lower than reported in a breast cancer cohort of similar age to the CREW sample.^33^ An overall lack of perceived social support was also reported in a cross‐sectional study of mixed cancer types in Korea, where levels of support were lower for colorectal cancer than for other cancers.^8^ In addition to considering the overall cohort, we also found distinct subgroups of participants, some of which had relatively stable levels of support over time (an estimated 71%), and others with poorer and declining support (21% mid/declining support and 8% low/declining support).

Most of the literature relating to change in social support following cancer diagnosis and treatment is in breast cancer, and to our knowledge no studies have described distinct subgroups. A study of colorectal cancer survivors in China (*N* = 227) reported levels of social support that were stable from pre‐surgery baseline to 3 months' follow‐up, declined up to 6 months, and remained low up to 1 year following surgery.^19^ A number of studies in different countries have reported decreased social support within the first 2 years following diagnosis and treatment for breast cancer, with some reporting changes as early as 6 months.^13^, ^14^, ^15^ Leung et al^5^ reported stability in social support ratings up to 3 years post‐diagnosis, although the number of women with 3 year data was low (*N* = 124 of the 412 in their study), and so they may have been unrepresentative. The cause of reduced perceived social support following cancer diagnosis and treatment is unclear. Eom et al^8^ suggested that prolonged caregiving burden and expectations of patients' recovery following cancer treatment may lead to a reduction in support from family and loved ones. In addition, individuals providing the support might not be aware what type of support is needed or indeed if it is still required as the post‐treatment time progresses, which suggests that carers themselves might need guidance on how best to provide support over time. Also, with patterns of follow‐up changing to earlier discharge, and hence less contact with health care professionals, this may have an impact on people's perception of levels of social support. It is therefore important to identify at diagnosis those most likely to have poorer and declining support so that relevant interventions can be put into place at an early stage.

### 4.2 Associations between participant characteristics and levels of social support

Our findings suggested that participants with lower and declining levels of social support were more likely to be older, female, with greater neighbourhood deprivation, to have co‐morbidities and rectal cancer. Those who could be regarded as most vulnerable and in need of support from others are those who might have fewer opportunities to interact with others or perceive less support available. Our findings are mostly consistent with previous findings, particularly with respect to poorer social support and lower socio‐economic status.^8^ A study of mixed cancer types in the US (including 380 colorectal cancer patients) reported higher perceived social support in those with adequate financial resources but no association with age or gender.^3^ Published evidence of gender differences in perceived social support varies. LeMasters et al^34^ reported lower levels of emotional support for women compared with men in around 2000 colorectal cancer patients in the USA, but a study of mixed cancer types in South Korea reported lower levels of perceived social support for men, although cultural differences may partly explain this finding.^8^ We found 2 clinical factors associated with poorer social support over follow‐up: having co‐morbidities and a stoma. Co‐morbidities have been shown to adversely affect HRQoL following cancer^35^ and may reduce social interactions, although conversely may increase support from health care professionals. Having a stoma has been reported as reducing participation in social activities and causing difficulties in relationships for colorectal cancer patients.^36^ Although we did not find an independent association between stoma and poorer perceived social support, participants with rectal cancer, where stomas are more common, reported poorer support compared with colon cancer.

### 4.3 Associations between levels of social support and HRQoL outcomes

Our findings confirmed published evidence that poorer social support is significantly associated with poorer HRQoL outcomes in colorectal cancer (independent of demographic and clinical factors).^11^, ^35^ A cohort study described a link between social support pre‐surgery for colorectal cancer and HRQoL outcomes including pain, fatigue, and social functioning at 12 months, although only 65% of the participants had follow‐up data and were different from those enrolled at baseline in a number of ways.^9^ Others have explored social networks, e.g., Sapp et al^10^ focused on colorectal cancer patients' social ties (number and frequency of contact) rather than perceived support and quality, and demonstrated the importance of social ties in maintaining or improving HRQoL, particularly mental health. The relationship between social support and depression is interdependent in that, just as anxiety and depression are related to a lower perception and availability of social support, poor social support in turn can lead to greater susceptibility to depression and anxiety. So, it is perhaps unsurprising that social support has been consistently shown to have a strong association with mental health in cancer survivors, including colorectal cancer.^6^, ^7^, ^8^, ^10^, ^12^, ^37^ Improved outcomes for patients with greater social support can also be interpreted within the context of promoting health care behaviours,^10^ such as adherence with follow‐up,^38^ and timing of commencing adjuvant chemotherapy^39^ as well as lifestyle factors.^40^

### 4.4 Strengths and limitations of the study

Strengths of our study include the size and representative nature of the CREW sample. Loss to follow‐up is generally inevitable in large cohort studies; however, response rates remained high up to 2 years (74%). The longitudinal design provided the opportunity to carry out repeated assessments at several time‐points evaluating a wide range of measures including HRQoL outcomes and a range of psychosocial domains including social support. The current paper reports 2 years' follow‐up, but data collection up to 5 years is ongoing, enabling further investigations into longer‐term outcomes following colorectal cancer treatment. The study also has the merits of including pre‐treatment baseline assessments. Regarding the assessment of social support, although we assessed actual and perceived support using a number of measures, we did not investigate details of social networks, including frequency of social ties and quality of support received from others that have been considered in previous studies,^10^, ^16^ or satisfaction with support.^7^ In addition, our study might have benefitted from measuring caregivers' perspectives on social support offered.

### 4.5 Clinical implications

Our study has important implications for how patients can be optimally supported throughout the disease and survivorship pathway to facilitate better recovery and outcomes. Assessments of levels of support at diagnosis in particular and throughout treatment and follow‐up would enable health care providers to identify vulnerable patients and put interventions in place to increase support from other sources where needed. This might involve signposting to support groups, invitations to health and wellbeing events, increasing patients' confidence in seeking and accepting support from others, and interventions to support carers. Our findings highlight the importance of asking about perceived levels of support; it cannot be assumed that for individuals who have a wide circle of friends and family, there is naturally a perception of high levels of support available to them. Health care professionals could ask patients to identify who they feel they can turn to for help or support when then need it. In addition, in view of our findings, this conversation should not just take place at diagnosis but be re‐visited throughout follow‐up.

## 5 CONCLUSIONS

A substantial proportion of people report poor and reduced social support following diagnosis and treatment for colorectal cancer. Social support is an important risk factor for recovery and HRQoL outcomes in colorectal cancer, particularly mental health. Vulnerable patients such as older people, those living in more deprived areas, and those with co‐morbidities have less social support and, therefore, need more targeted professional support throughout their care, with social support implemented from the point of diagnosis as part of a holistic approach to health care provision.

## CONFLICT OF INTEREST

None.

## AUTHOR CONTRIBUTIONS

JH and SS wrote the paper, with contributions and comments from LC, JC, AD, DF, CG, AR, PWS, JW, and CF. JH performed the statistical analyses.

## ETHICS STATEMENT

All procedures performed in studies involving human participants were in accordance with the ethical standards of the institutional and/or national research committee and with the 1964 Helsinki Declaration and its later amendments or comparable ethical standards.

## ORIGINAL PUBLICATION STATEMENT

The manuscript contains original unpublished work and is not being submitted for publication elsewhere at the same time.

## Supporting information

Appendix S1: Medical Outcomes Study—Social Support Survey used in CREW questionnaires

Appendix S2 Table A1: Associations between baseline characteristics and estimated trajectories of social support up to 2 years

## References

[1] Bloom JR , Petersen DM , Kang SH . Multi‐dimensional quality of life among long‐term (5+ years) adult cancer survivors. Psychooncology. 2007;16(8):691‐706. 17628036 10.1002/pon.1208

[2] Foster C , Fenlon D . Recovery and self‐management support following primary cancer treatment. Br J Cancer. 2011;105 Suppl 1(S1):S21‐S28. 22048029 10.1038/bjc.2011.419 PMC3251956

[3] Forsythe LP , Alfano CM , Kent EE , et al. Social support, self‐efficacy for decision‐making, and follow‐up care use in long‐term cancer survivors. Psychooncology. 2014;23(7):788‐796. 24481884 10.1002/pon.3480 PMC4082440

[4] Ferreira DB , Koifman R , Bergmann A . Quality of life in Brazilian women with breast cancer: association with the social environment. J Epidemiol Commun H. 2011;65:A241‐AA41.

[5] Leung J , Pachana NA , McLaughlin D . Social support and health‐related quality of life in women with breast cancer: a longitudinal study. Psychooncology. 2014;23(9):1014‐1020. 24700668 10.1002/pon.3523

[6] Al‐Ahwal MS , Al Zaben F , Khalifa DA , Sehlo MG , Ahmad RG , Koenig HG . Depression in patients with colorectal cancer in Saudi Arabia. Psychooncology. 2015;24(9):1043‐1050. 25328130 10.1002/pon.3706

[7] Costa ALS , Heitkemper MM , Alencar GP , Damiani LP , da Silva RM , Jarrett ME . Social support is a predictor of lower stress and higher quality of life and resilience in Brazilian patients with colorectal cancer. Cancer Nurs. 2017;40(5):352‐360. 27171810 10.1097/NCC.0000000000000388

[8] Eom CS , Shin DW , Kim SY , et al. Impact of perceived social support on the mental health and health‐related quality of life in cancer patients: results from a nationwide, multicenter survey in South Korea. Psychooncology. 2013;22(6):1283‐1290. 22833521 10.1002/pon.3133

[9] Gonzalez‐Saenz de Tejada M , Bilbao A , Bare M , et al. Association of social support, functional status, and psychological variables with changes in health‐related quality of life outcomes in patients with colorectal cancer. Psychooncology. 2016;25(8):891‐897. 26582649 10.1002/pon.4022

[10] Sapp AL , Trentham‐Dietz A , Newcomb PA , Hampton JM , Moinpour KM , Remington KL . Social networks and quality of life among female long‐term colorectal cancer survivors. Cancer. 2003;98(8):1749‐1758. 14534893 10.1002/cncr.11717

[11] Dunn J , Ng SK , Breitbart W , et al. Health‐related quality of life and life satisfaction in colorectal cancer survivors: trajectories of adjustment. Health Qual Life Outcomes. 2013;11:46 23497387 10.1186/1477-7525-11-46 PMC3648454

[12] Dunn J , Ng SK , Holland J , et al. Trajectories of psychological distress after colorectal cancer. Psychooncology. 2013;22(8):1759‐1765. 23125004 10.1002/pon.3210

[13] Salonen P , Tarkka MT , Kellokumpu‐Lehtinen PL , Huhtala H , Kaunonen M . Effect of social support on changes in quality of life in early breast cancer patients: a longitudinal study. Scand J Caring Sci. 2013;27(2):396‐405. 22834764 10.1111/j.1471-6712.2012.01050.x

[14] Thompson TRT , Perez M , Schootman M , Jeffe DB . Perceived social support change in patients with early‐stage breast cancer and controls. Health Psychol. 2013;32(8):886‐895. 23477582 10.1037/a0031894 PMC3935244

[15] Lee MK , Park S , Lee ES , et al. Social support and depressive mood 1 year after diagnosis of breast cancer compared with the general female population: a prospective cohort study. Support Care Cancer. 2011;19(9):1379‐1392. 20676695 10.1007/s00520-010-0960-4

[16] Fong AJ , Scarapicchia TM , McDonough MH , Wrosch C , Sabiston CM . Changes in social support predict emotional well‐being in breast cancer survivors. Psychooncology. 2017;26(5):664‐671. 26818101 10.1002/pon.4064

[17] Song L , Northouse LL , Braun TM , et al. Assessing longitudinal quality of life in prostate cancer patients and their spouses: a multilevel modeling approach. Qual Life Res. 2011;20(3):371‐381. 20927648 10.1007/s11136-010-9753-y PMC3888242

[18] Penedo FJ , Traeger L , Benedict C , et al. Perceived social support as a predictor of disease‐specific quality of life in head‐and‐neck cancer patients. J Support Oncol. 2012;10(3):119‐123. 22088826 10.1016/j.suponc.2011.09.002

[19] Hong CJ‐LT , Lin C , Ben‐Qiang X , Shaojun Z , Xiao‐Bing T , En‐Quan Z . The temporal decline of social support among colorectal cancer survivors: first year prospective study. Iran J Public Health. 2016;45(10):1368‐1368. PMC5149502 27957445

[20] Fenlon D , Richardson A , Addington‐Hall J , et al. A cohort study of the recovery of health and wellbeing following colorectal cancer (CREW study): protocol paper. BMC Health Serv Res. 2012;12:90 22475242 10.1186/1472-6963-12-90 PMC3382420

[21] Office for National Statistics 2015 Retrieved from https://www.gov.uk/government/statistics/english-indices-of-deprivation-2015

[22] Sherbourne CD , Stewart AL . The MOS social support survey. Soc Sci Med. 1991;32(6):705‐714. 2035047 10.1016/0277-9536(91)90150-b

[23] The EuroQol Group . EuroQol—a new facility for the measurement of health‐related quality of life. Health Policy. 1990;16(3):199‐208. 10109801 10.1016/0168-8510(90)90421-9

[24] The International Wellbeing Group . Personal Wellbeing Index—Adult (PWI‐A). 5th ed. Melbourne: The Australian Centre on Quality of Life, Deakin University; 2013 http://www.deakin.edu.au/research/acqol/instruments/wellbeing-index/index.php.

[25] Spielberger CD , Gorsuch RL , Lushene R , Vagg PR , Jacobs GA . Manual for the State‐Trait Anxiety Inventory (STAI). Palo Alto, CA: Consulting Psychologists Press; 1983.

[26] Radloff LS . The CES‐D scale: a self report depression scale for research in the general population. Appl Psychol Measur. 1977;1:385‐401.

[27] Knight RG , Waalmanning HJ , Spears GF . Some norms and reliability data for the State Trait Anxiety Inventory and the Zung Self‐Rating Depression Scale. Br J Clin Psychol. 1983;22(Nov):245‐249. 6640176 10.1111/j.2044-8260.1983.tb00610.x

[28] Katz MR , Kopek N , Waldron J , Devins GM , Tomlinson G . Screening for depression in head and neck cancer. Psychooncology. 2004;13(4):269‐280. 15054731 10.1002/pon.734

[29] Hardin JW , Hilbe JM . Generalized Estimating Equations. Second ed. *Chapman & Hall/CRC*; 2013 *ISBN‐13: 978‐1‐4398‐8113‐2* 2013.

[30] Jones BL , Nagin DS , Roeder K . A SAS procedure based on mixed models for estimating developmental trajectories. Sociol Method Res. 2001;29(3):374‐393.

[31] Schwarz G . Estimating the dimension of a model. Ann Stat. 1978;6(2):461‐464.

[32] Foster C , Haviland J , Winter J , et al. Pre‐surgery depression and confidence to manage problems predict recovery trajectories of health and wellbeing in the first two years following colorectal cancer: results from the CREW Cohort Study. PLoS One. 2016;11(5): e0155434 10.1371/journal.pone.0155434 PMC4865190 27171174

[33] Jatoi A , Muss H , Allred JB , et al. Social support and its implications in older, early‐stage breast cancer patients in CALGB 49907 (Alliance A171301). Psychooncology. 2016;25(4):441‐446. 25994447 10.1002/pon.3850 PMC4864498

[34] LeMasters T , Madhavan S , Sambamoorthi U , Kurian S . A population‐based study comparing HRQoL among breast, prostate, and colorectal cancer survivors to propensity score matched controls, by cancer type, and gender. Psychooncology. 2013;22(10):2270‐2282. 23606210 10.1002/pon.3288 PMC4892175

[35] Jansen L , Koch L , Brenner H , Arndt V . Quality of life among long‐term (≥5 years) colorectal cancer survivors—systematic review. Eur J Cancer Care. 2010;46(16):2879‐2888. 10.1016/j.ejca.2010.06.010 20605090

[36] Liao C , Qin Y . Factors associated with stoma quality of life among stoma patients. Int J Nurs Sci. 2014;1(2):196‐201.

[37] Mosher CE , Winger JG , Given BA , Helft PR , O'Neil BH . Mental health outcomes during colorectal cancer survivorship: a review of the literature. Psychooncology. 2016;25(11):1261‐1270. 26315692 10.1002/pon.3954 PMC4894828

[38] Le D , Holt CL , Pisu M , et al. The role of social support in posttreatment surveillance among African American survivors of colorectal cancer. J Psychosoc Oncol. 2014;32(3):245‐263. 24611486 10.1080/07347332.2014.897293 PMC5568546

[39] Malietzis G , Mughal A , Currie AC , et al. Factors implicated for delay of adjuvant chemotherapy in colorectal cancer: a meta‐analysis of observational studies. Ann Surg Oncol. 2015;22(12):3793‐3802. 25777086 10.1245/s10434-015-4479-2

[40] Kroenke CH , Michael YL , Shu XO , et al. Post‐diagnosis social networks, and lifestyle and treatment factors in the After Breast Cancer Pooling Project. Psychooncology. 2017;26(4):544‐552. 26749519 10.1002/pon.4059 PMC4938778

