# Supplementary figures and images for: Social support following diagnosis and treatment for colorectal cancer and associations with health‐related quality of life: Results from the UK ColoREctal Wellbeing (CREW) cohort study
Source: Psychooncology. 2017 Nov 1;26(12):2276–84. doi: 10.1002/pon.4556 (PMC6220760; doi:10.1002/pon.4556)

**Appendix 1: Medical Outcomes Study - Social Support Survey used in CREW questionnaires**
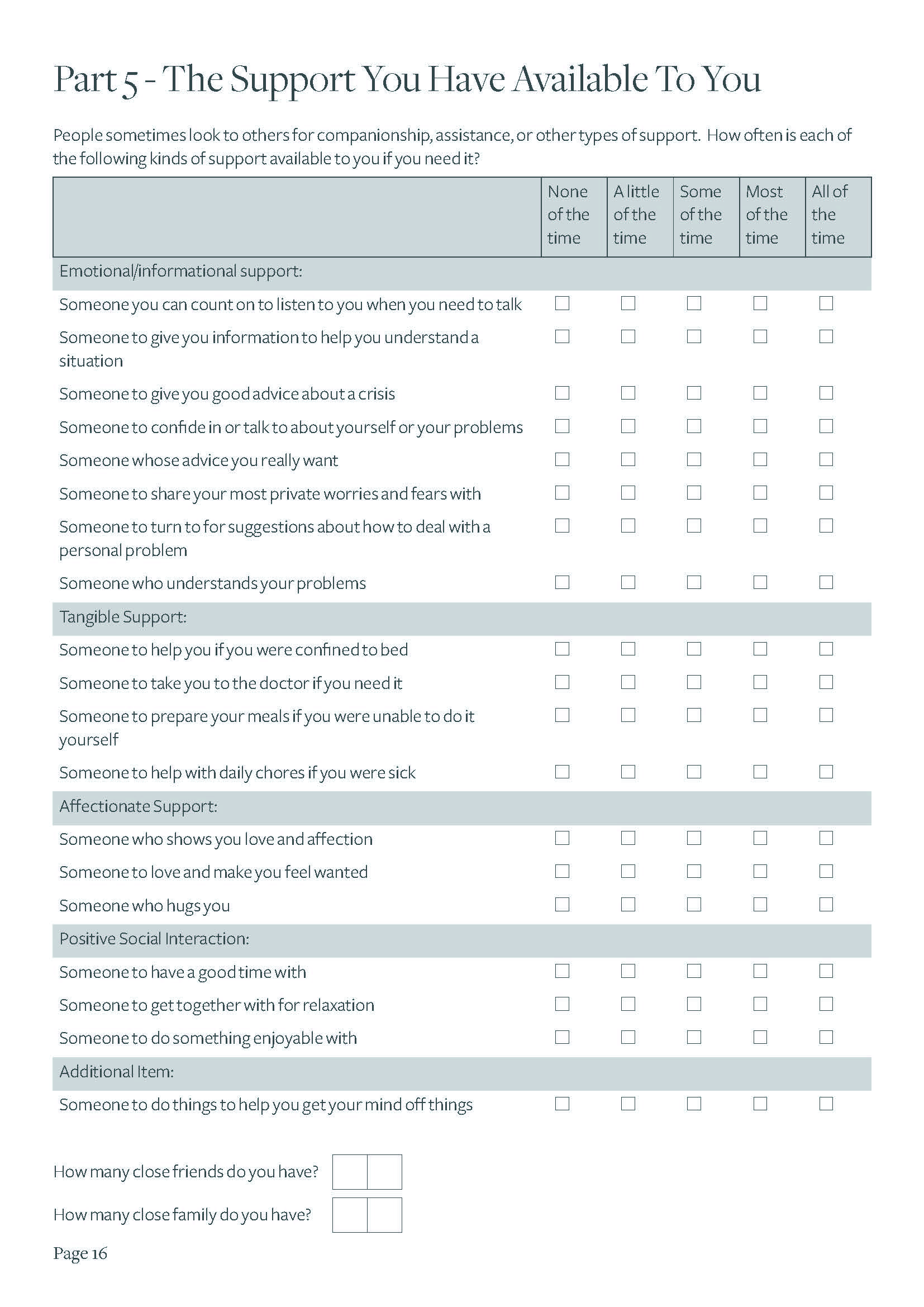

Supplement: Supplementary file 1 — Appendix S1: Medical Outcomes Study—Social Support Survey used in CREW questionnaires [file PON-26-2276-s001.docx]
