# Supplementary material for: Social support following diagnosis and treatment for colorectal cancer and associations with health‐related quality of life: Results from the UK ColoREctal Wellbeing (CREW) cohort study
Source: Psychooncology. 2017 Nov 1;26(12):2276–84. doi: 10.1002/pon.4556 (PMC6220760; doi:10.1002/pon.4556)
Supplement: Supplementary file 2 — Appendix S2 Table A1: Associations between baseline characteristics and estimated trajectories of social support up to 2 years [file PON-26-2276-s002.docx]

**Appendix 2**

**Table A1: Associations between baseline characteristics and estimated trajectories of social support up to 2 years**

| **Baseline characteristics** | **Adjusted odds ratio^1^ (95%CI) for each trajectory group relative to Group 1 of MOS-SSS overall social support, p-value** | | | |
| --- | --- | --- | --- | --- |
|  | **Group 1 (very high & constant)**  **N=268** | **Group 2 (good & constant)**  **N=308** | **Group 3**  **(mid & declining)**  **N=172** | **Group 4**  **(low & declining)**  **N=60** |
| **Age**  Older | Ref. group  1 | p=0.392  0.89 (0.68-1.16) | p=0.046  1.32 (1.00-1.73) | p=0.460  1.16 (0.78-1.72) |
| **Gender**  Female vs male | Ref. group  1 | p=0.423  1.21 (0.76-1.93) | p=0.046  1.64 (1.01-2.67) | p=0.278  1.49 (0.72-3.08) |
| **Neighbourhood deprivation quintile**  1^st^ (least deprived), 2^nd,^ 3^rd,^ 4^th,^ 5^th^ (most deprived) | Ref. group  1 | p=0.138  1.13 (0.96-1.33) | p=0.114  1.15 (0.97-1.36) | p=0.049  1.30 (1.00-1.69) |
| **Any co-morbidities***  Yes vs no | Ref. group  1 | p=0.176  1.45 (0.85-2.49) | p=0.822  0.94 (0.55-1.60) | p=0.021  3.65 (1.22-10.95) |
| **Tumour site**  Rectum vs colon | Ref. group  1 | p=0.190  1.41 (0.84-2.35) | p=0.995  1.00 (0.57-1.77) | p=0.013  2.57 (1.22-5.41) |

^1^ Odds ratios adjusted for all variables in table and pre/post-surgery baseline. Age and deprivation quintile fitted as continuous covariates, so odds ratios represent odds of group membership relative to Group 1 per unit increase in characteristic. P-values from Wald test comparing each Group with Group 1 (reference group) in multiple regression model.

* Co-morbidities reported on 3-month questionnaire
